# Inhibition of HSV-1 Replication by Gene Editing Strategy

**DOI:** 10.1038/srep23146

**Published:** 2016-04-11

**Authors:** Pamela C. Roehm, Masoud Shekarabi, Hassen S. Wollebo, Anna Bellizzi, Lifan He, Julian Salkind, Kamel Khalili

**Affiliations:** 1 Department of Neuroscience, Center for Neurovirology, Temple University School of Medicine, 3500 N. Broad Street, Philadelphia, 19140 PA, USA; 2 Department of Otolaryngology/Head and Neck Surgery, Temple University School of Medicine, 3500 N. Broad Street, Philadelphia, 19140 PA, USA; 3 Department of Neurosurgery, Temple University School of Medicine, 3500 N. Broad Street, Philadelphia, 19140 PA, USA.

## Abstract

HSV-1 induced illness affects greater than 85% of adults worldwide with no permanent curative therapy. We used RNA-guided CRISPR/Cas9 gene editing to specifically target for deletion of DNA sequences of the HSV-1 genome that span the region directing expression of ICP0, a key viral protein that stimulates HSV-1 gene expression and replication. We found that CRISPR/Cas9 introduced InDel mutations into exon 2 of the ICP0 gene profoundly reduced HSV-1 infectivity in permissive human cell culture models and protected permissive cells against HSV-1 infection. CRISPR/Cas9 mediated targeting ICP0 prevented HSV-1-induced disintegration of promonocytic leukemia (PML) nuclear bodies, an intracellular event critical to productive HSV-1 infection that is initiated by interaction of the ICP0 N-terminus with PML. Combined treatment of cells with CRISPR targeting ICP0 plus the immediate early viral proteins, ICP4 or ICP27, completely abrogated HSV-1 infection. We conclude that RNA-guided CRISPR/Cas9 can be used to develop a novel, specific and efficacious therapeutic and prophylactic platform for targeted viral genomic ablation to treat HSV-1 diseases.

Herpes simplex virus type 1 (HSV-1) is a human neurotropic virus that infects the majority of the human population worldwide^1^,^2^. The HSV-1 genome is present in 85–90% of trigeminal ganglia of individuals at autopsy^3^, and its seroprevalence is 90% in normal asymptomatic individuals^4^. In most cases, after initial infection HSV-1 persists in only a latent stage, without causing obvious symptoms. However, in some cases the primary infection causes fever, dysphagia, odynophagia, and painful blisters on the lips and tongue^1^,^5^, and in neonates, primary HSV-1 infection can lead to significant morbidity and mortality^1^,^6^. Replication of HSV-1 in the nervous system causes encephalitis with symptoms ranging from fever, focal deficits, aphasia and seizures resulting from the infection and anti-viral inflammatory response within the frontal lobe and the temporal lobe^7^. The site of HSV-1 latency appears mainly restricted to cranial nerve ganglia, although in some cases the viral genome has been detected in thoracic ganglia and brain^8^,^9^. Current treatments for primary HSV-1 infection and reactivation of diseases are non-selective, do not prevent establishment of latent infection or viral reactivation, and have adverse side effects, pointing to a strong need for improved and specific therapeutic strategies.

The mechanisms of HSV latency initiation and persistence are not fully understood, but evidence points to a role of viral RNA elements (latency-associated transcripts; LATS), which are abundantly expressed during latency^10^,^11^,^12^,^13^. Environmental factors including UV light stimulation, hyperthermia, social stress and a host of pharmacological agents can trigger reactivation of the latent HSV-1 genome, productive viral replication in neurons, and disease progression. The most common and easily identifiable clinical sign of HSV-1 reactivation is cold sores (herpes labialis), but other conditions including recurrent genital herpes have been linked to HSV reactivation^2^.

Current anti-HSV drugs include viral DNA synthesis (*e.g*. thymidine kinase; TK) inhibitors. Such drugs can diminish spread of HSV-1 infection to other cells and limit the extent of damage, but fail to inhibit latency establishment or HSV reactivation. Future reactivation events can only be impeded by chronic administration antiherpetic drugs, which induce development of drug-resistant HSV-1 strains. Patients, particularly those with underlying immunodeficiency, are at risk for developing HSV-1 strains resistant to TK inhibitors, subsequently requiring generic treatments including DNA inhibitors that are not HSV-1-specific, and thus elicit a range of adverse side effects. Also, TK inhibitors are associated with renal toxicity. Attempts to develop a vaccine against HSV-1 have been relatively ineffective^1^,^14^.

Given the limitations of current therapy, there is a clear need for new treatment avenues to permanently eliminate virus or disable its reactivation in latent cells, and effectively suppress viral replication in lytically infected cells. Studies of protective responses of single-cell organisms including *Streptococcus pyogenes* against invasion by bacteriophage and transposable DNA elements led to discovery of the CRISPR/Cas9 system^15^. The endogenous type II CRISPR system in such microbes apparently defends against foreign DNA, as association of CRISPR RNA (crRNA) and transactivated RNA (trRNA) sequences can trigger targeting of foreign DNA sequences for double-strand cleavage by the DNA endonuclease Cas9. The specificity of such cleavage is determined by complementary base pairing between crRNA and sequences on the target DNA^16^. Exploiting this, the Cas9 system has recently been modified to permit specific genomic editing in mammalian cells. Upon entering the nuclei of eukaryotic cells and utilizing the guide RNAs (gRNAs), Cas9 introduces insertion/deletion (InDel) mutations into target genes^17^. CRISPR/Cas9 has been used to modify several eukaryotic genes, and several groups have explored its antiviral potential^17^,^18^,^19^,^20^,^21^,^22^,^23^. To assess its translational potential for application to treat and eliminate HSV-1 infection, we tested the ability of CRISPR/Cas9 to suppress HSV-1 replication by targeting specific DNA sequences essential to viral protein expression during early and late phases viral infection/reactivation.

During primary HSV-1 infection, a defined class of sequentially expressed viral proteins direct a successful productive infection cycle. After viral entry into cells, viral capsid proteins are released within the cytoplasm, causing shutdown of host protein synthesis. Also during this immediate early phase of viral infection, 100–200 copies of infected cell protein 0 (ICP0), which is associated with capsid proteins, is transported to the inner tegument and facilitates nuclease-mediated entry of capsid proteins to the sites where viral replication occurs^24^. Concurrently, the viral gene encoding ICP0 is activated, and newly synthesized ICP0 promotes efficient progression of lytic infection, which is more similar to real-life infection than high dose infection models. Therefore, ICP0 contributes more effectively to viral survival and replication during low-dose viral infection. ICP0 is also important in HSV-1 reactivation and has critical roles in lytic infection, including disruption of PML bodies and avoidance of innate immune responses^25^,^26^,^27^. Therefore, we targeted DNA sequences of the ICP0 gene and developed guide RNAs to combine with Cas9, to test their ability to suppress viral gene expression. We found this strategy introduced specific InDel mutations in the ICP0 sequence, produced no off-target effects or toxicity to host cells, and rescued antiviral PML bodies from disintegration by ICP0, supporting the promise of this model approach for developing anti-HSV-1 therapies capable of eliminating latent infection.

## Results

### gRNA Targeting, Construction and testing in Vero cells

**Figure 1:**
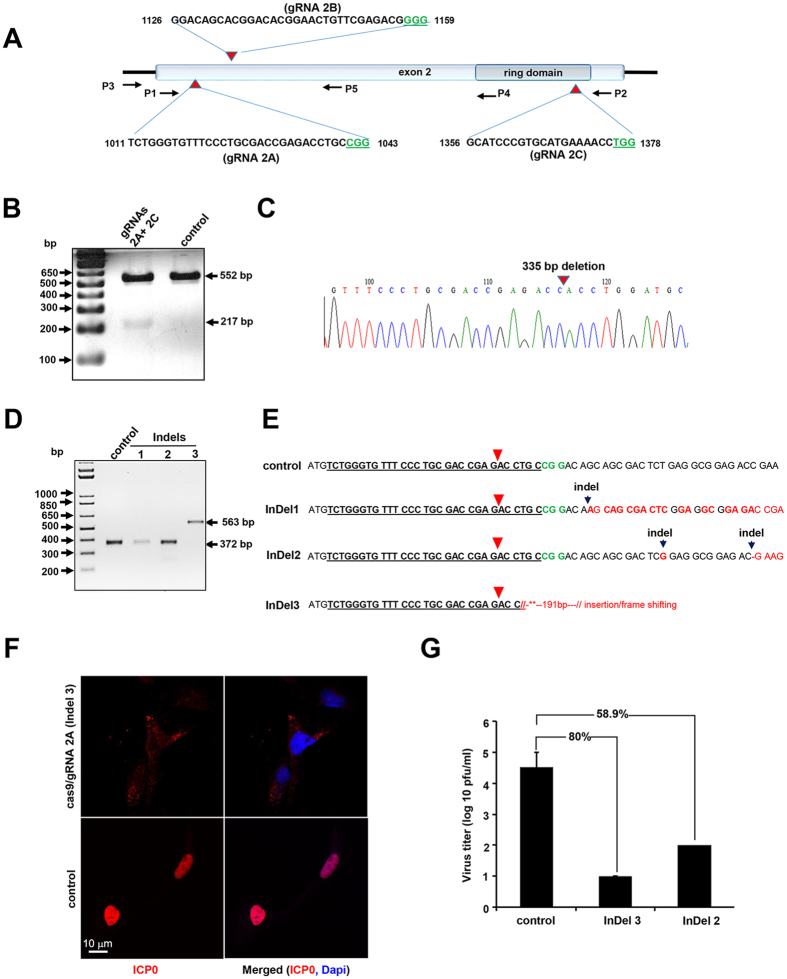
CRISPR/Cas9 introduces mutations in ICP0 gene and reduces HSV-1 replication. (**A**) Schematic representation of HSV-1 ICP0 genomic region showing exon II and the ring domain. The positions and nucleotide composition of 2A, 2B and 2C targets including PAM (marked in green) are shown. The positions of various primers (P) used in PCR amplification are illustrated. (**B**) Gel analysis of DNA fragments amplified by primers P3 and P2 (shown in A) in L7 cells expressing Cas9 (control) or Cas9 plus gRNAs 2A and 2C. The positions of an expected 552 bp amplicon and a smaller DNA fragment of 217 bp caused by cleavage of exon II DNA at targets (**A**,**C**), and amplification of the remaining fragments after ligation are shown. (**C**) Sequence traces of the 217 bp cloned fragment (shown in **B**) illustrating a blunt end-joining of the two ends of the cleaved exon II DNA after excision of 355 bp. D. DNA gel analysis illustrating amplicons using P3 and P4 primers from several clonal cells (named InDels 1-3) expressing Cas9/gRNA 2A or the control cells with no gRNA 2A. (**E**) DNA sequencing identified nucleotide insertions (as depicted by black arrowheads) in clones InDel 1 and 2, and insertion of 191 bp DNA in clones InDel 3. The red arrowhead points to the cleavage sites. (**F**) Representative confocal images of L7 cells (control) and its subclones InDel 3 (shown in **E**) expressing Cas9/gRNA 2A after immunostaining with an antibody against ICP0. Green is indicative of expression of GFP by HSV-1 and DAPI (blue) depicts the nuclei of the cells. (**G**) Viral production assay demonstrating the titer of ΔICP0-HSV-1 in the supernatant of parental L7 cells (control) endogenously expressing ICP0 or in InDel 3, InDel 2 (shown in **E**).

As noted above, ICP0 is an important immediate early regulatory protein of the herpes simplex virus with the greatest impact on expression of the viral gene and its replication^26^. Furthermore, it has been shown that ICP0-negative viruses are avirulent and immunogenic, suggesting a potential role for the inhibition of HSV spread and regulation of viral latency^28^,^29^. As such, we have selected the ICP0 gene as a potential target for editing by the CRISPR/Cas9 strategy. Bioinformatic screening of the HSV-1 genome identified three specific regions within exon II of the ICP0 gene, spanning nucleotide (nt) sequences 1011–1043 (2A), 1126–1159 (2B) and 1356–1378 (2C), for creating guide RNAs (gRNAs) for editing the ICP0 gene by CRISPR/Cas9 (Fig. 1A). Small DNA fragments corresponding to each motif were first cloned separately in expression vector PX260^18^, and their abilities to induce InDel mutations, when expressed in single form, or to excise a DNA fragment spanning between the cleavage sites, when used in combination, were assessed by PCR amplification using specific primers as shown in Fig. 1A and Table S1 followed by Sanger sequencing. Initially we used an ICP0-complementing L7 cell line^27^, herein called L7, which contained an integrated copy of the ICP0 gene and expressed low levels of the encoded protein when the cells are infected with HSV-1 for transfection with Cas9 along with gRNA 2A- and 2C-expressing plasmids. In addition to an expected 552 bp amplicon, a smaller DNA fragment of 217 bp was also detected in cells expressing both gRNAs 2A and 2C (Fig. 1B). We verified excision of a 335 bp DNA fragment spanning between the 2A and 2C sites by Sanger sequencing, and amplification of a smaller (217 bp) DNA fragment using a template created by re-joining the remaining DNA of exon II.

Further analyses in clonal cell lines showed that in a small population of cells (8%), removal of a 217 bp DNA fragment was accompanied by insertion of a single A or G nucleotide at the cleavage and rejoining sites (Fig. S1A). Similarly, multiplex expression of gRNA 2B and 2C caused excision of a 219-bp DNA fragment between 2B and 2C, and amplification of a 251-bp DNA fragment (Fig. S1B). In single-gRNA expression configuration, expressing gRNA 2A produced several InDel mutations, detected by Cel1 nuclease-based heteroduplex specific SURVEYOR assay (Fig. S1C). Characterization of the InDel mutations by DNA sequencing of several clonal cells revealed insertion of single or multiple single nucleotides, as well as insertion of a large 191-bp DNA clone close to the cleavage sites (Fig. 1D,E). As noted, each of these mutations caused a frameshift in the ICP0 DNA coding sequences (Fig. 1E). We compared the expression of ICP0 in control L7 cells to that in clone InDel 3 upon infection with HSV-1 harboring a reporter GFP gene. As shown in Fig. 1F (lower panels), infection of control cells with HSV-1/GFP induced nuclear accumulation of ICP0, where newly replicated virus is present. In contrast, Cas9/gRNA 2A prevented detectable ICP0 appearance within the nuclei of infected cells, indicating that the frameshift mutations introduced in exon II of the ICP0 completely blocked expression of the protein and its subcellular localization (Fig. 1F, upper panels). ICP0 (red) was co-localized in the cytoplasmic areas, suggesting that Cas9/gRNA has also caused InDel mutations in the input HSV-1 DNA, thus eradicating expression of ICP0 from the incoming virus.

We also assessed the impact of these mutations on the ability of ICP0 to stimulate HSV-1 replication, by comparing ΔICP0 HSV-1 replication levels in these clones (InDel 2 and InDel 3) to those seen in control cells expressing the wild-type proteins. Supernatants of each infected cell culture were collected 48 h after infecting cells with ΔICP0 HSV-1, and virus titer was determined by plaque assay. Clones InDel 2 and InDel 3 showed marked declines in ability to support replication of ΔICP0 HSV-1 (58.9% and 80%, respectively), indicating Cas9/gRNA 2A-mediated functional inactivation of ICP0 in L7-cells (Fig. 1G). Production of gRNA and Cas9 was verified by RT-PCR and Western blot analysis, respectively (Fig. S2).

### Impact of Cas9/gRNA on PML nuclear body structure

Promyelocytic leukemia (PML) nuclear bodies are punctate structures localized within nuclei of many eukaryotic cells that control cell growth and transformation^30^), and also inhibit infection of cells by many viruses including HSV-1^31^,^32^. Interaction of ICP0 with HSV-1 with PML via its N-terminus apparently suppresses PML-mediated anti-viral activity, by disintegrating PML structure^31^,^33^.

**Figure 2:**
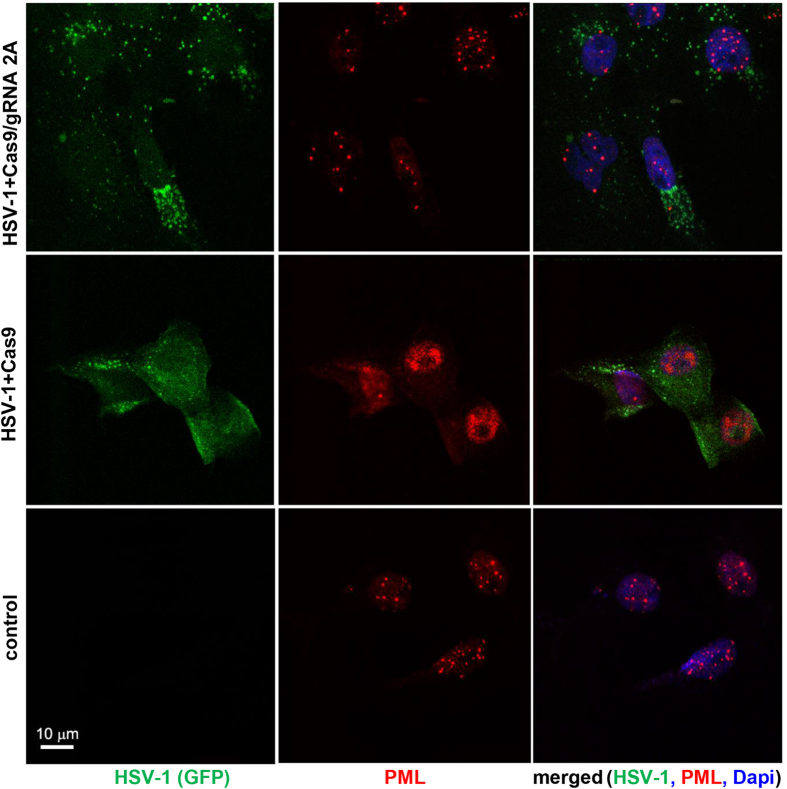
CRISPR/Cas9 by targeting ICP0 expression restores association of PML bodies in L7 cells. Immunostaining analysis of PML bodies in control, uninfected L7 cells shows the appearance of punctate staining of PML bodies within the nuclei of the cells (lower panel). DAPI staining of nuclei (blue) is shown in the right panels. Infection of Vero L7 cells expressing Cas9 at low MOI with HSV-1/GFP shows complete destruction of the PML and robust replication of HSV-1/GFP as shown in green (middle panels). In L7 cells expressing Cas9/gRNA 2A infection with HSV-1/GFP failed to disrupt formation of punctate PML in the nuclei and significantly reduced the level of viral replication detected in the cells (green, top panels).

PML-associated nuclear bodies punctate structures were evident by fluorescent microscopy in the nuclei of HSV-1-uninfected L7 cells expressing only Cas9 (Fig. 2), but infection of these cells with HSV-1/GFP completely changed the structure of PML punctate structures coincident with HSV-1 localization. In contrast, co-expression of both Cas9 and gRNAs in infected L7 cells restored the pattern of PML-like punctate structures to one very similar to that seen in uninfected cells. Low levels of GFP were also detected in the cytoplasm, probably representing expression of GFP encoded by the viral genome in the absence of its productive infection cycle (Fig. 2). This observation suggests that Cas9/gRNA-induced mutation of ICP0 interferes with its PML body-disrupting antiviral activity.

### Expression of Cas9/gRNA suppresses HSV-1 infection of human cell line

**Figure 3:**
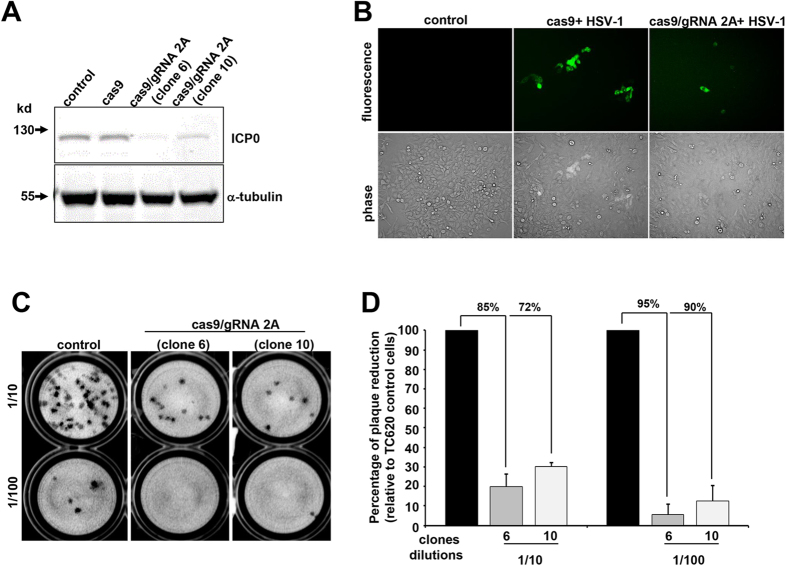
Human TC620 cells expressing Cas9/gRNAs does not support HSV-1 replication. (**A**) Western blot analysis of protein extracts for detection of ICP0 in TC620 human olidodendroglioma cells and its subclones expressing Cas9 or Cas9/gRNA 2A (clones 6 and 10). Expression of 110 kDa ICP0 is detected in the control cells and cells expressing Cas9, but severely reduced in clones 6 and 10. Expression of housekeeping tubulin is shown. (**B**) Representative plaque assay using the supernatant from HSV-1/GFP infected control TC620 or clones 6 and 10 at two different dilutions showing a drastic decrease in the number of plaques as a result of suppression of ICP0 by Cas9/gRNA editing of the infected cells. (**D**) Quantification of HSV-1 production by plaque assay shows drastic suppression of the viral production in TC620 cells with continuous production of Cas9 plus gRNA 2A.

Seeking a convenient human cell line in which to further explore the anti-HSV-1 activity of our CRISPR/Cas9 system, we found that the human oligodendroglioma cell line TC620^34^ robustly supported HSV-1 replication at low and high multiplicities of infection, as evidenced by expression of ICP0, the early viral protein ICP8, which is involved in viral DNA replication and late gene transactivation^35^,^36^,^37^, and the late envelope protein, glycoprotein C, which enhances infectivity of the virus^38^ (Fig. S3A–D). We created several clonal TC620 cell sublines that stably expressed Cas9 or Cas9 plus gRNA 2A, and used them to test the ability of Cas9/gRNA 2A to suppress HSV-1 infection. The clonal lines 6 and 10, where both Cas9 and gRNAs are produced, showed a substantial decrease in the level of ICP0 production, seen in Western blots, whereas cells that only express Cas9 did not show any affect in the ICP0 production level (Fig. 3A). We confirmed by fluorescence microscopy for HSV-1/GFP that Cas9/gRNA 2A expression suppressed HSV-1 infection of TC620 cells. We compared supernatants collected after HSV-1 infection from control cells expressing only Cas9, and from clones 6 and 10, using plaque assay with Vero cells at 1/10 and 1/100 dilutions (Fig. 3C). We found that Cas9/gRNA 2A suppressed plaque formation in clones 6 and 10, respectively, by 85% and 72% at 1/10 dilution, and by 95% and 90% at 1/100 dilution (Fig. 3C,D). The enhanced suppression at the higher dilution concords with prior findings that the inhibitory effects of ICP0 are more pronounced at lower MOI infection^39^. These data indicate that in uninfected cells, Cas9/gRNA 2A protects cells against HSV-1 infection, by inhibiting ICP0 production and hence its suppression of host antiviral defenses, contributing to lytic infection.

We also assessed the potential effects of Cas9/gRNA 2A on several indices of cellular health status. Expression of Cas9, either alone or together with gRNA 2A, had no significant effect on cell cycle progression of TC620 cells (Fig. S4A). Similarly, immunofluorescent staining of cells with annexin indicated no major impacts of Cas9 or Cas9/gRNA 2A on apoptosis (Fig. S4B). Accordingly, we saw no adverse effects of the gene-editing system on cell viability (Fig. S4C). To evaluate the specificity of gRNA 2A and determine whether our gene editing strategy induced any off-target effects or compromises host genome integrity, we used the SURVEYOR assay. The results from gel analysis showed no evidence for InDel mutations in any potential exonic off-target sites in several representative human genes that were identified by bioinformatic screening using a shorter (13 nt seed sequence corresponding to the 2A sequence of HSV-1 (Fig. S5A, also see Table S2). Further, results from gene amplification using a pair of specific primers (shown in Table S3) followed by DNA sequencing of the potential cellular off-targets genes, as noted above, confirmed no off-target effects of the HSV-1 aimed gene editing system (Fig. S5B).

### Lentivirus mediated delivery of Cas9 and gRNA suppresses HSV-1 infection and protects cells from infection

**Figure 4:**
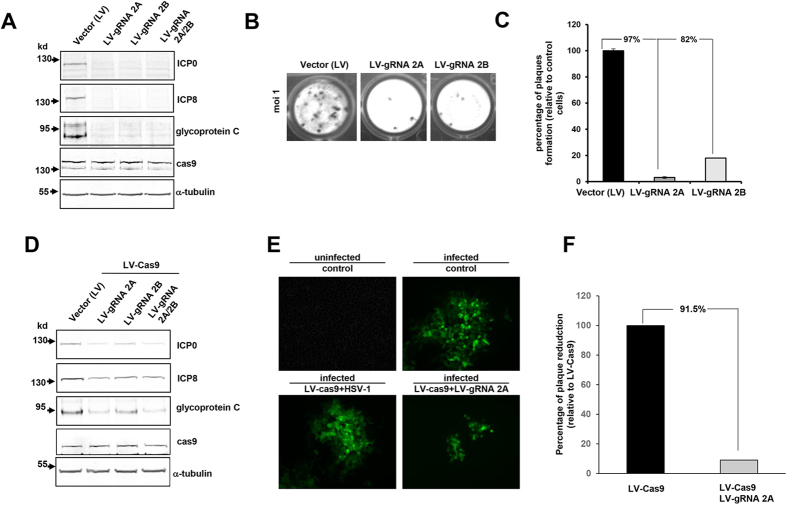
Lentivirus (LV) mediated Cas9/gRNA delivery suppresses HSV-1 infection during the lytic cycle and prevents uninfected cells from new infection. (**A**) Western blot analysis of protein extracts from TC260 cells that endogenously express Cas9 and are infected with HSV-1/GFP for 24 hours and thereafter treated with lentivirus expressing gRNAs 2A, 2B or both and harvested 48 hours later for protein extraction. The positions of ICP0, ICP8, glycoprotein C Cas9 and α-tubulin are shown. (**B**,**C**) illustrate results from plaque assay in which cells treated with LVgRNA 2A or LVgRNA 2B showed a drastic decrease in virus production. (**D**) Confocal immunofluorescent evaluation of HSV-1/GFP replication in TC620 cells that were treated with vector (LV) LVCas9, LVCas9 plus LVgRNA 2A, LVCas9 plus gRNA 2B and LVCas9 plus LVgRNA 2A and 2B at 48 hours prior to HSV-1 infection. A decrease in the replication of HSV-1/GFP is shown in green. (**D**) Western blot analysis of protein extracts from the infected cells as described above. (**E**) Plaque assay showing a decrease in the titer of the virus released upon HSV-1 infection of cells that were pre-treated with LV producing Cas9 and gRNAs as depicted.

To further improve delivery efficiency and evaluate anti-HSV-1 activity of our gene editing molecules, TC620 cells expressing Cas9 were infected with HSV-1 and 36 hours later, HSV-1 infected cells were transduced with lentivirus (LV) expressing gRNA 2A or 2B in single or duplex configuration. Results showed markedly suppressed expression of ICP0, ICP8, and the late glycoprotein C (Fig. 4A), indicating that the incoming gRNAs by lentivirus suppressed HSV-1 protein production in Cas9-expressing cells. Treating the cells with LVgRNA 2A and LVgRNA 2B, drastically suppressed viral production of infected cells (to 97% and 82%, respectively), as measured by plaque assay of infectious virus in culture supernatants (Fig. 4B,C). Throughout these studies, we noticed that gRNA 2A is slightly more effective than gRNA 2B in editing the ICP0 gene and suppressing HSV-1 replication. To assess the ability of Cas9 and gRNAs 2A, 2B or both in protecting cells from HSV-1 infection, we transduced normal TC260 cells with lentivirus expressing Cas9 (LVCas9), along with lentivirus expressing gRNAs (LVgRNAs) 2A, 2B, or both. We found that LVCas9/LVgRNA 2A significantly decreased viral protein expression (Fig. 4D) confirmed by the results from fluorescence microscopy showing great suppression of GFP stained cells (Fig. 4E) and a drastic decline in viral abundance as evaluated by plaque assay (Fig. 4F).

ICP4 and ICP27 are the two other immediate early (IE) regulatory proteins largely responsible for the transition from the IE to the early phase of viral gene transcription, and regulating viral and cellular mRNA processing events and activation of late genes, respectively^27^,^40^. In the next series of experiments, after bioinformatic screening of the ICP4 and ICP27 genes, we selected gRNAs with no prediction of off-target effects (Table S4) and assessed their ability to collaboratively suppress HSV-1 infection of TC260 cells. As shown in Fig. S6, lentivirus-mediated delivery of ICP0 gRNA ICP4 gRNA ICP27 gRNA reduced the extent of HSV-1 infection of TC260 cells (compare Panel A with Panel B, top). Interestingly, we observed no residual infected cells when combinations of gRNAs targeting more than one viral protein were used in this study (Fig. S6, compare top panel and bottom panel in Panel B).

## Discussion

Current therapy to suppress HSV-1 replication includes nucleoside analogues that target the viral thymidine kinase (TK), including acyclovir and its derivatives. Such agents help control the symptoms of primary HSV-1 infection, but do not eliminate latent HSV-1 infection. Further, development of HSV-1 strains that are resistant to TK inhibitors, due to mutations in the TK genes, remains a clinical challenge in the treatment of HSV-1 associated illness.

We tested the ability of CRISPR/Cas9 system to permanently suppress HSV-1 infection by altering the viral genes responsible for the immediate early phage of the infection cycle. We chose ICP0 as our target for editing as it controls the balance between HSV-1 latency and replication and plays an important role in blocking several cell-mediated anti-viral activities including PML nuclear bodies^40^. Moreover, ICP0 of HSV-1 is highly sensitive to inhibition by interferon α and β, and elicits strong immune responses against wild-type HSV-1^28^. Thus, abrogating ICP0 expression can indirectly interfere with replication of new HSV-1 infection. Our findings indicate that targeting the ICP0 gene using CRISPR/Cas9, induced multiple InDel mutations in exon II of ICP0, halted disruption of the endogenous antiviral PML assemblies, and completely abrogates the viral life cycle in the infected cells.

Among the three gRNAs that we identified for ICP0 gene targeting by CRISPR/Cas9, gRNA 2A showed the greatest potency in single configuration to compromise the structure of exon II of the ICP0 genome by inducing InDel mutations that led to the generation of frameshift in the ICP0 open reading frame. This mutation in exon II also interfered with ICP0 interaction with PML and formation of nuclear bodies. Thus, Cas9/gRNA 2A exerts its antiviral activity by inhibiting expression of multiple HSV-1 genes whose products are required for the infection cycle and halting of host-derived anti-viral events including the assembly of PML bodies. Lentivirus-mediated delivery of Cas9 and gRNAs targeting ICP0 during viral infection significantly decreased viral loads produced by infected cells. Our results from combinatory experiments using ICP0 gRNA plus either ICP4 gRNA or ICP27 gRNA showed a drastic reduction of HSV-1 replication in the infected cells. This preliminary observation suggests that elimination of HSV-1 at the latent and productive stages may require editing of multiple viral genes, an approach that is achievable considering the simplicity and flexibility of the CRISPR/Cas9 strategy. The combined strategy also mitigates concern related to repair of one hit or possible recombination, should a HSV-1 based delivery system be used. Further, we found that Cas9 expression in cells prior to infection suppressed HSV-1 replication when gRNA was introduced to the cells by lentivirus, suggesting that the Cas9 strategy will be applicable to protecting cells against HSV infection. In this respect, one can envision development of a novel therapeutic strategy in which Cas9 gene only becomes activated by a HSV-1 immediate early protein such as ICP4, thus allowing Cas9 to remain silent in the absence of HSV-1 and reactivated only when the virus begins to enter the lytic phase of infection.

The prospects for translation of Cas9/CRISPR-based viral targeting to human clinical therapies depend not only upon development of efficient delivery systems, but also upon their specificity. As a first step to assessing this critical issue in our HSV-targeting system *in vitro*, we surveyed its effects on key cell functions, indices of cell health, and potential genomic targets. We found our Cas9/gRNA system lacks adverse effects upon cell viability and processes including including the cell cycle, does not induce apoptosis, and does not jeopardize cell viability. To achieve this level of safety, avoid effects on human translated genomic sites and exclude any transcription DNA motif, we employed bioinformatic screening based on the strictest 12 bp plus PAM target selection criteria. Our most effective gRNA (2A) was 30 nt in length plus NGG. It showed no evidence for InDel mutation in any of a series of five representative off-target host genes identified by bioinformatic screening using shorter (12 nt) seed sequences corresponding to target A of ICP0 exon II. Our results, also show that the CRISPR/Cas9 system can be refined and used for protecting cells against HSV-1 infection. The pre-existence of Cas9 plus gRNAs 2A in human cells that robustly support HSV-1 replication prevented efficient replication of the incoming HSV-1. This observation is reminiscent of the strategy by which prokaryotic cells protect themselves from foreign DNA elements such as transposable elements and bacteriophages^41^. Given the ease and rapidity of the CRISPR/Cas9 gene editing system and the complexity of HSV-1 lytic infection cycle, a combination therapy can be developed that includes a cocktail of gRNAs for targeting important viral proteins involved in the regulation of the immediate early, early and late phases of HSV-1 infection. For delivering Cas9/gRNAs, there are several virus-based systems available including lentivirus, AAV, modified HSV-1 vectors. Non-viral delivery systems including nanoparticles and extracellular vesicles may also be used. In addition, expression of Cas9 can be modulated through various inducible expression systems to control its expression or to expedite Cas9 turnover, which helps to limit off-target effects. One other consideration relates to the effect of antiviral drugs such as Acyclovir, as the latently infected individuals are usually under antiviral therapy. Our preliminary results have shown that antiviral treatments have no effect on the CRISPR gene editing, thus it is expected that cessation of antiviral therapy after successful implantation of CRISPR/Cas9, no rebound in viral replication will be detected. Currently, studies are in progress to demonstrate complete elimination of HSV-1 under various conditions in latently infected neuronal culture and in *in vivo* systems.

In summary, our findings demonstrate that our Cas9/gRNA editing system targeting ICP0 introduced mutations in this immediate early gene of HSV-1 with high specificity, suspends the HSV-1 lytic infection cycle, and protects uninfected cells from HSV-1 infection. Our results show that introducing a single nucleotide mismatch in the ICP0 coding sequence by our gene editing strategy is highly effective in blocking viral replication. Thus, our studies provide new evidence that CRISPR/Cas9 can be developed as a new therapeutic tool for HSV-1 infection and a potential and permanent strategy for eliminating HSV-1 DNA from latently infected cells.

## Materials and Methods

### Cell Culture

ICP0-complementing L& cell line L7^27^ was grown in Dulbecco’s Modified Eagle’s Medium (DMEM) (Life Technologies, NY) supplemented with 10% fetal bovine serum (FBS), 2 mM glutamine and 400 μg/ml of Geneticin (Life Technologies, NY). Normal Vero cells were grown in the same medium without Geneticin. The human oligodendroglioma cell line TC620 were maintained in DMEM supplemented with 10% FBS as we have previously described^22^. For selection, cells were transfected with appropriate vectors and selected using the above medium containing 3 μg of puromycin (Life Technologies) for 5 days when they were replated onto 96 cell well plates at 0.5 cell per well in the presence of puromycin. In order to produce clonal derivatives of TC620 cells expressing Cas9 and gRNAs, this cells were transfected with pX260 or pX260-derived plasmids expressing each of the gRNA that are described below. Selection was done with 3 μg/ml puromycin and clones isolated by dilution cloning. All transfections were performed using Lipofectamine 2000 (Life Technologies) according to the manufacturer protocol.

Cell viability assay was performed using Live/Death cell viability assay kit (Molecular probes, NY) according to the manufacturer protocol on Vero or L7 cells transfected with different anti-ICP0 gRNAs after three days.

### Antibodies

The following antibodies were used for the Western blot. Mouse α-ICP0 (1:1000, Santa Cruz, TX), Mouse monoclonal [11E2] ICP8 (1:1000, Abcam), Mouse monoclonal [3G9] α-gC (1:1000, Abcam) Mouse anti-Flag M2 (1:1000, Sigma Aldrich), anti-tubulin clone B512 from (1:5000, Sigma Aldrich)

### Apoptosis Assay

Apoptosis assay was performed according to manufacturer’s recommendation (Guava Technologies). Briefly, the control and Cas9 stable expressing cells were plated in 6 well plates at density of 200,000 cells/ml for 48 hrs. Before harvesting the cells, the supernatant of each sample is collected in 15 ml falcon tube and the cells were washed with PBS and trypsinized with 0.25% trypsin. Trypsin was inactivated with the supernatant collected above for each sample. The samples were centrifuged for 5 min at 2000 rpm. The supernatant was aspirated and the pellet was washed with PBS once and the cells were resuspended and counted and diluted to density of 100,000 cells/ml in PBS. 100 μl of room temperature Annexin V-PE staining reagent was mixed with 100 μl of cell suspension and incubated for 25 min at room temperature in the dark. Samples were analyzed using a Guava Easy Cyte mini flow Cytometer.

### Cell cycle analysis

Control and Cas9 stably expressing cells were plated at density of 150,000 cells/ml for 48 hrs. Cells were collected and harvested by centrifugation. The pellet was washed once with PBS and resuspended in 1 ml PBS and fixed in ice cold ethanol (70% final concentration) . The cells were incubated for 24 hours at −20 °C. The cells were centrifuged, pelleted, washed once with PBS containing 1% BSA and stained with propidium iodide (10 μg/ml) in PBS containing 250 μg/ml RNase A and incubated at 37 °C for 45 min in the dark. Cell cycle analysis was performed with the Guava Easy Cyte mini system using the Guavasoft cell cycle program.

### Viability assay

For viability assay, the control and Cas9 stably expressing cells were plated in triplicate in 96 well plates at a density of 12000 cells/200 μl for 24 hrs. Two hours before the MTT assay, the old media was removed and replaced with the new one. The cells were treated with 20 μl of MTT solution (5 mg/ml MTT in PBS) for 2 hours in 37 °C. After 2 hours, the media was removed and replaced with 200 μl of MTT solvent (4 mM HCl, 0.1% Nonidet P-40 (NP-40) in isopropanol) and the cells were put on a shaker for 10 min. MTT activity was measured at channels 590 nm and 620 nm.

### HSV-1 ICP0 gRNAs

The genomic sequence of HSV-1 ICP0 (NC_0018061) was obtained from NCBI database and *ICP0* exon 2 open reading frame was determined using the published ICP0 protein sequence and NCBI database. Three gRNAs were designed and selected using available online tools (http://crispr.mit.edu/). CRISPR Cas9 off target finding tools were used to determine off target sequences.

A pair of DNA oligos from each target sequence were designed in forward and reverse orientations based on published and recommended flanking sequences for pX458 and pX260 vectors (Addgene plasmids 48138 and 42229, respectively). Each pair were annealed in a thermocycler, using 5 μl of each oligo at the concentration of 100 nM at 95 °C for 7 mins and ramped at 3% from 95 °C to 25 °C in the presence of 2 μl of T4 DNA ligase buffer and 9 μl of water in 20 μl of total reaction. Annealed oligo pairs were then cloned into BbsI linearized pX458 and pX260 vectors. The insertion of the gRNAs were confirmed using sequencing. Plasmid preps were prepared using Plasmid Midi kit (Qiagen).

### InDel mutation analysis

Deletions and/or insertions of nucleotides in exon 2 of HSV-1 ICP0 were verified by single or co-transfection experiments in L7 cells. Two micrograms of pX260 (carrying puromycin resistant gene and ICP0 gRNA 2A or 2B) and PX458 constructs (carrying EGFP-Cas9 and ICP0 gRNA 2C to estimate the transfection efficiency) were cotransfected for overnight. L7 cells were transfected with empty vectors were served as control. The transfection efficiency was estimated using an inverted fluorescence microscope by examining multiple fields and counting GFP positive cells versus total cell number. Genomic DNA extractions were performed using Archive PureDNA extraction kit (5Prime, MD) after three days. Genomic DNA amplifications were performed using Q5 Hot Start High-Fidelity DNA polymerase (NEB, MA) and two primers which were designed from ICP0 sequence to amplify a fragment with 552 bp or 470 bp in size (shown in Table S1) using following conditions; 98 °C (30s) then 35 cycles with 98 °C (10s), 62 °C (30s), 72 °C (30s) and then 72 °C (5 min). The PCR products were analyzed on a 3% agarose gel. The bands of interest were gel-purified and cloned into pCRII T-A vector (Life Technologies), and the nucleotide sequence of individual clones was determined by sequencing at Genewiz.

### RT-PCR

To determine the expression of gRNA 2A in L7 and TC620 stable cells lines, RT-PCR was performed essentially as described previously^42^, with the exception of using a pX260 based reverse primer (pX260-crRNA-3′, Table 1) as a primer to generate the cDNAs. To amplify crRNA, the same primer was paired with the gRNA 2A forward oligo.

### ICP0^558^-Ds-Red Construct

To generate ICP0^558^-Ds-Red, two primers were designed with Age I restriction sites on each ends and used to amplify a 558 bp fragment from the exon 2 of HSV-1 ICP0 genome using Q5 Hot Start (NEB) using ICP0-AgeI-F (3086–3103, NC_0018061) and ICP0-AgeI-R (3626–3643, NC_0018061) (shown in Table S1). pDs-Red-C1 and the amplified fragment were digested with Age I and ligated using T4 DNA ligase (NEB) resulting in a final in-framed with the Ds-Red protein sequence. After selection, the cells were plated onto 96 well plates at the density of 0.5 cells/well to increase the probability one cell per well seeding. The cells were then allowed to grow to 60–70% confluence when they were cotransfected with EGFP and ICP0^558^-Ds-Red constructs using Lipofectamine 2000. 48 hours post transfection the cells were examined under an inverted fluorescence microscope and wells with no red signals were selected for further analysis. Genomic DNA was prepared and PCR-amplified using P3 and P4 primers spanning a 372 bp fragment consisting of 2A target sequence (Table S1). The amplified fragments were gel purified using Qiaex II gel extraction kit (Qiagen) and sequenced to verify InDel sequence.

### Immunocytochemistry and Western blots

Two L7 clones were picked and plated onto 8 well chamber slides at 6000 cells per well. They were then infected with 5 MOI of HSV-1 (Patton strain) GFP-Us11 for 2 hours. At 6 hours post infection, the cells were quickly rinsed with cold Balanced Salt Solution (HBSS, Invitrogen) before fixation with 4% paraformaldehyde. The cells were immunostained essentially as described elsewhere^43^. Mouse α-ICP0 (0.4 μg/mL, Santa Cruz, TX), and mouse anti-PML C7 (2 μg/mL, Abcam, MA) antibodies were used for immunostaining at 4 °C for overnight. Alexa Fluor 555 secondary anti-mouse antibody (Molecular Probes; Invitrogen), were used (1:1,000) to visualize mouse primary antibodies. The nuclei were labeled with 0.5 μg/ml Hoechst 33258 (Sigma, MO) in PBS for 30 min. Our observations were carried out using a Leica TCS SP5 broadband confocal microscope equipped with the AOBS (acousto-optical beam splitter) for optimal beam splitting.

For protein analysis of TC620 cells, whole cell extracts were prepared in TNN buffer containing mammalian protease inhibitor cocktail (Sigma Aldrich). 50 μg of protein was separated by 10% SDS-PAGE, transferred to nitrocellulose. Blots were blocked in 5% milk in 1XPBST and immunoblotted with primary antibodies (1:1000) for 2 hours at room temperature. After washing, the blots were incubated with secondary antibody Goat Anti-Mouse (1:5000) LI-COR dyes and visualized with an Odyssey CLx Imaging system (LI-COR, INC, Lincoln, NE) using LI-COR Odyssey software.

### Viral titer

To assay the infection efficiency HSV-1 on gRNA 2A clonal cells, we first infected the mock transfected L7 cells or G9 (InDel 1) or H5 (InDel 2) with one MOI of wild type or ICP0 mutant HSV-1 strain^44^ for two hours at 37 °C in MEM medium (Life Technologies). We then collected and titered the supernatant containing newly generated viral particles by infecting normal Vero cells using serial dilutions. After 24 or 48 hours cells were fixed in 10% Trichloroacetic acid (Sigma) for 10 minutes and stained with 0.05% of Crystal violet (Sigma). The plaques were counted and graphed.

### Infection of TC620 cells with HSV-1

One day before infection, we plated 45,000 TC620 cells per well in 48 wells plate with DMEM supplemented with 10% FBS. On the day of infection, we aspirated the media from TC620 cells and added 100 μL of diluted virus media into each well. After 2 hours of incubation at 37 °C, aspirated media containing virus and added fresh TC620 media back on. Cells were incubated for 2 days.

### Production of lentiviral vectors for gRNAs and transduction of TC620 cells stably expressing Cas9

To construct the gRNA lentiviral expression plasmids for each of the two targets, the U6 expression cassette from each of the two pX330 gRNA plasmids that were described above was amplified by PCR using primers (5′-TATGGGCCCACGCGTGAGGGCCTATTTCCCATGATTCC-3′ and 5′-TGTGGATCCTCGAGGCGGGCCATTTACCGTAAGTTATG-3′). The PCR products were cut with MluI and BamH1 and then cloned into pKLV-U6gRNA (BbsI)-PGKpuro2ABFP that had been cut with MluI and BamH1. The pCR™4-TOPO^®^ TA vector was from Life Technologies, Inc., (Carlsbad, CA). To produce lentiviral vectors for transduction of the two gRNAs, each of the two gRNA lentiviral expression plasmid derivatives constructed as described above from pKLV-U6gRNA(BbsI)-PGKpuro2ABFP were transfected into 293T cells by calcium chloride precipitation together with packaging plasmids pCMV-VSV-G, pMDLg/pRRE and pRSV-Rev. Lentivirus was harvested from the supernatant after 48 h, cleared by centrifugation and passage through a 0.45 μm filter and added to TC620 cells stably expressing Cas9 in the presence of 6 μg/ml polybrene followed by selection. After 48 hours the cells were harvested and analyzed for ICP0 expression by Western blot and viral titer by plaque assay.

### SURVEYOR assay

The presence of mutations in PCR products of off-target genes derived from TC620 cells stably expressing Cas9 and transduced by lentiviral vectors for gRNAs was examined using the SURVEYOR Mutation Detection Kit (Transgenomic) according to the manufacturer’s protocol. Heterogeneous PCR product was denatured for 10 min at 95 °C and then hybridized by gradual cooling using a thermocycler. Three hundred nanograms of hybridized DNA (9 μl) was digested with 0.25 μl of SURVEYOR Nuclease, which is a mismatch-specific DNA endonuclease used to scan for mutations in heteroduplex DNA, plus 0.25 μl SURVEYOR Enhancer S and 15 mM MgCl2 for 4 h at 42 °C. Stop Solution was added and samples were resolved on a 2% agarose gel together with equal amounts of control samples treated in parallel but derived from TC620 cells stably expressing Cas9 but not transduced by lentiviral vector for gRNA.

## Additional Information

**How to cite this article**: Roehm, P. C. *et al*. Inhibition of HSV-1 Replication by Gene Editing Strategy. *Sci. Rep.* **6**, 23146; doi: 10.1038/srep23146 (2016).

## Supplementary Material

Supplementary Information
