# Supplementary material for: Inhibition of HSV-1 Replication by Gene Editing Strategy
Source: Sci Rep. 2016 Apr 11;6:23146. doi: 10.1038/srep23146 (PMC4827394; doi:10.1038/srep23146)
Supplement: Supplementary Information [file srep23146-s1.pdf]

2/4/16

SUPPLEMENTARY MATERIALS

Inhibition of HSV-1 Replication by Gene Editing Strategy

Running Title: Targeting HSV-1 Infection by CRISPR/Cas9

Pamela C. Roehm[1,2,3*], Masoud Shekarabi[1], Hassen Wollebo[1],

Anna Bellizzi[1], Lifan He[1], Julian Salkind[1], Kamel Khalili[1*]

1. Department of Neuroscience, Center for Neurovirology; 2. Department of Otolaryngology/Head and Neck Surgery; 3. Department of Neurosurgery; Temple University School of Medicine, 3500 N. Broad Street, Philadelphia, PA 19140

Keywords: CRISPR/Cas9, herpes simplex type 1, DNA editing, ICP0, PML Nuclear Body

M.S. and H.W. contributed equally to this work.

*Corresponding authors:

Kamel Khalili kamel.khalili@temple.edu (senior corresponding author)

Pamela Roehm, pamela.roehm@tuhs.temple.edu

Phone: 215-707-5192/Fax: 215-707-4888

Table S1: Primers (HSV-1)

Primer	Sequence
P1	5'-GATGTCTGGGTGTTTCCCT-3'
P2	5'-CACGATCGGGATGGTGCTGAACGACC-3'
P3	5'- TGCTGATTGACGCGGGAAATC-3'
P4	5'- CGATCTCATCCGTGCACACG-3'
P5	5'-CCATCCACGCCCTGCGGCCCCAGC-3'

Table S2: Predicted ICP0 gRNAs and their off-target numbers (100% match)

	gRNA sequence	20	19	18	17	16	15	14	13	12
gRNA 2A	1. TCCCTGCGACCGAGACCTGCCGG	0	0	0	0	0	0	0	7	19
gRNA 2B	2. ACACGGAACTGTTCGAGACGGGG	0		0	0	0	0	0	0	0
gRNA 2C	3.GCATCCCGTGCATGAAAACCTGG	0	0	0	0	0	0	0	8	31

The genomic sequence of HSV-1 ICPO (NC_ 0018061) was used to search for Cas9/gRNA target sites containing a 20-bp guide sequence plus the protospacer adjacent motif sequence (NGG) using Jack Lin's CRISPER/Cas9 finder tool(http://spot.colorado.edu/slin/cas9.html)

NGG(AGG,TGG,GGG,CGG) was blasted against available human genomic and transcript sequence with 1000 aligned sequences being displayed. After pressing Control+F, copy/paste the Target sequences (1-23 through 9-23 nucleotides) and find the number of genomic targets with 100% match. The number of off-targets for each searching was divided by 3 because of repeated genome library. The number shown indicates the sum of four search (NGG). The red nubmer indicates the gRNAs target sequences farthest from NGG

Table S3: Primers (cellular genes)

Primer	Sequence
off-1 (G32)	5'-CACGTCTGTGGGTGTTCAGA-3'<br>5'-AAAGCTCCTAGGGCTGTTGG-3
off-2 (G42)	5'-GGAACCCCCTCGCTTTCTTT-3'<br>5'-CGTATGTGTCCCATCGGCAT-3'
off-3 (G22)	5'-CACTGCCTTGTGCCCATAGA-3'<br>5'-TTGAAGCTGCACGTGTTGGA-3'
off-4 (G12)	5'-ACTGACGGGATCTGGGATCA-3'<br>5'-TTCACTGGGAGACGTCAAGC-3'
off-5 (G6)	5'-GTGAGGCCCCACACATGATT-3'<br>5'-AAGGATGAGAGATGGGGAAGC-3'

Table S4: Sequence of guide RNAs targeting HSV genes

Target genes	Sequence
ICP4 m1	5'-TTCTACGCGCGCTATCGCGACGG-3'
ICP27 m1	5'-GGAGTGTTCCTCGTCGGACGAGG-3'

SUPPLEMENTARY FIGURE LEGENDS

Figure S1. A. Chromatograms showing the detection of breakpoints within exon II as a result of cleavage of DNA by Cas9 and gRNAs 2A and 2C, and insertion of a single nucleotide A (top) and G (bottom) at the site of the breakpoint. B. Recombination of gRNA 2B and 2C cuts within exon II generating a new template for amplification of a 251 bp DNA. C. SURVEYOR assay showing InDel mutation in exon II at target A causing a new DNA fragment of 180 bp after cleavage with Cel1 nuclease.

Figure S2. A. Production of gRNA 2A in L7 cells (control) expressing Cas9 and Cas9 plus gRNA 2A by RT-PCR. β-actin transcripts served as a loading control. B. Expression of flag-Cas9 in TC620 cells were verified by Western blot analysis.

Figure S3. A. TC620, a human olidodendroglioma cell line, were infected with HSV-1/GFP at MOI of 0.1, 1 or 2, and 48 hours later, expression of GFP, indicative for HSV-1 expression and replication, was assessed by fluorescence microscopy. B. Western blot analysis for evaluating expression of ICP0 in TC620 cells infected with HSV-1 at 48 hours post-infection. C and D. Detection of ICP8 and glycoprotein C in infected TC620 cells at MOI=1, 48 hours post-infection by Western blot analysis.

Figure S4. A. Cell cycle assay was used to investigate the impact of Cas9/gRNA on the cell cycle of TC620 control cells has no expression of Cas9 or any gRNAs, whereas subclones of Cas9 and Cas9/gRNA 2A clones 6 and 10 express either Cas9 alone or Cas9 plus gRNA2A as indicated. As seen, no drastic differences in cell cycle progression through G1, S and G2/M phases were detected in these cell lines. B. Apoptotic assay in the clonal cell lines as shown in Panel A showed no significant changes in annexin stained cell population (ranging from 1.0-

2.1%). C. Cell viability assay using propidium iodide staining assay showed changes in the viability of the cells with Cas9 or Cas9/gRNA expression in the various clones.

Figure S5. A. DNA gel analysis of SURVEYOR assay illustrating no detectable bands below the expected DNA fragment from PCR amplification that can be attributed to gRNA 2A expression. Control is provided by the supplier of the SURVEYOR assay kit (Integrated DNA Technologies, Coralville, IA). B. Results from PCR amplification and Sanger sequencing of the cellular genes that contained potential off-targets (as shown in Panel A) revealed no InDel mutations in the amplicons. Arrows demonstrate the positions of the primers and their distance from the potential off-targets. Red indicates the PAM sequence and the block illustrate the potential target sequence for each cellular gene. The size of each amplicon is indicated.

Figure S6. Epifluorescent assessment of HSV-1/GFP replication in TC260 cells after treatment with lentivirus vector (LV) expressing Cas9 (Panel A) or Cas9 plus various gRNAs as shown above each panel according to the method described for Fig. 4E. Images are representative of duplicate plates for each condition.

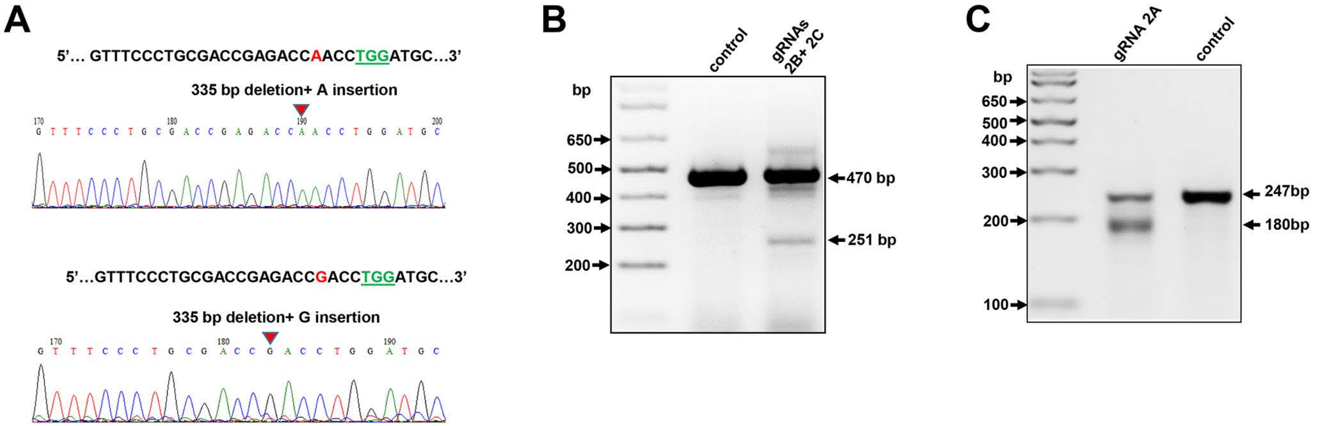

A
5'… GTTTCCCTGCGACCGAGACCAACCTGGATGC…3'
335 bp deletion+ A insertion
5'…GTTTCCCTGCGACCGAGACCGACCTGGATGC…3'
335 bp deletion+ G insertion
B
control
gRNAs 2B+ 2C
bp
650
500
400
300
200
470 bp
251 bp
C
gRNA 2A
control
bp
650
500
400
300
200
100
247bp
180bp

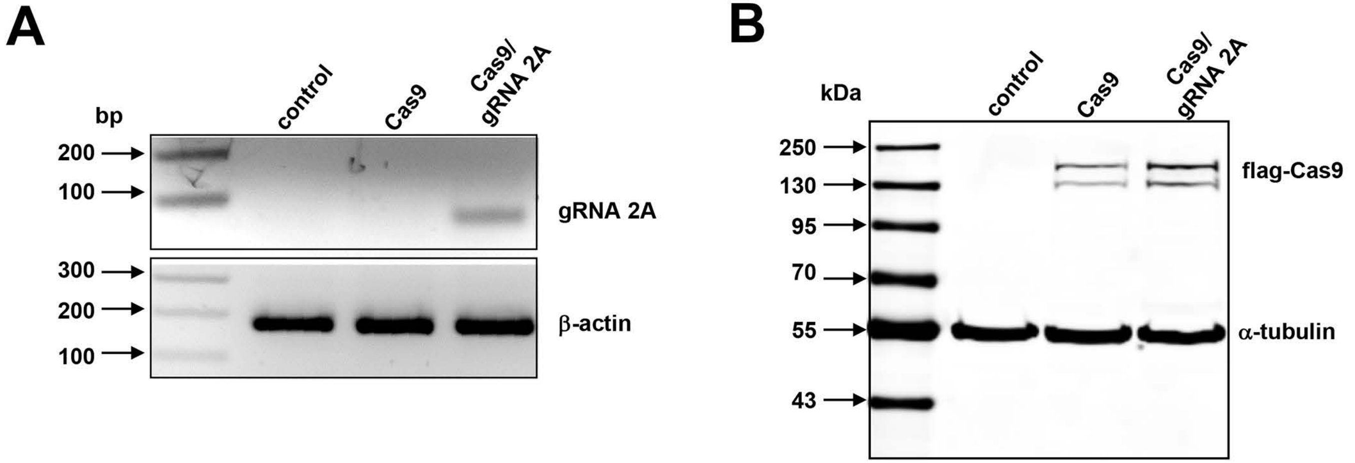
A
bp
control
Cas9
Cas9/
gRNA 2A
200
100
gRNA 2A
300
200
100
β-actin
B
kDa
control
Cas9
Cas9/
gRNA 2A
250
130
95
70
55
43
flag-Cas9
α-tubulin

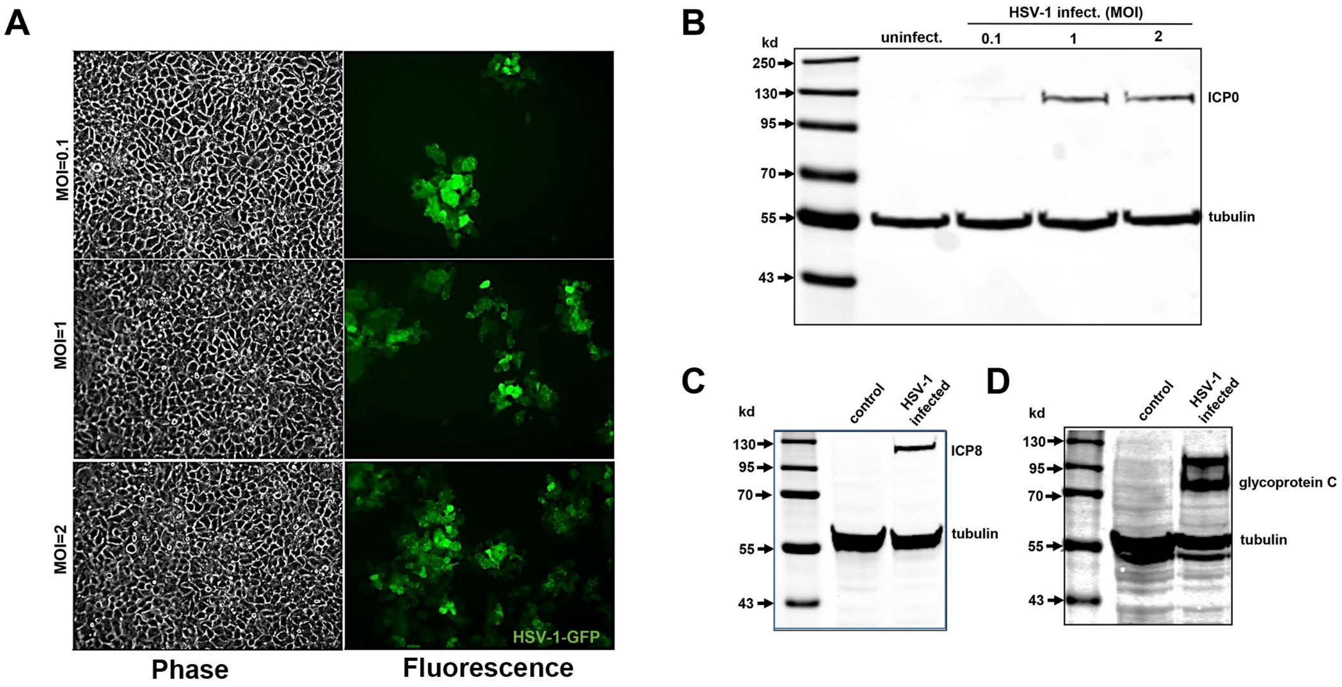

A
MOI=0.1
MOI=1
MOI=2
HSV-1-GFP
Phase
Fluorescence
B
HSV-1 infect. (MOI)
kd
uninfect.
0.1
1
2
250
130
95
70
55
43
ICP0
tubulin
C
kd
control
HSV-1 infected
130
95
70
55
43
ICP8
tubulin
D
kd
control
HSV-1 infected
130
95
70
55
43
glycoprotein C
tubulin

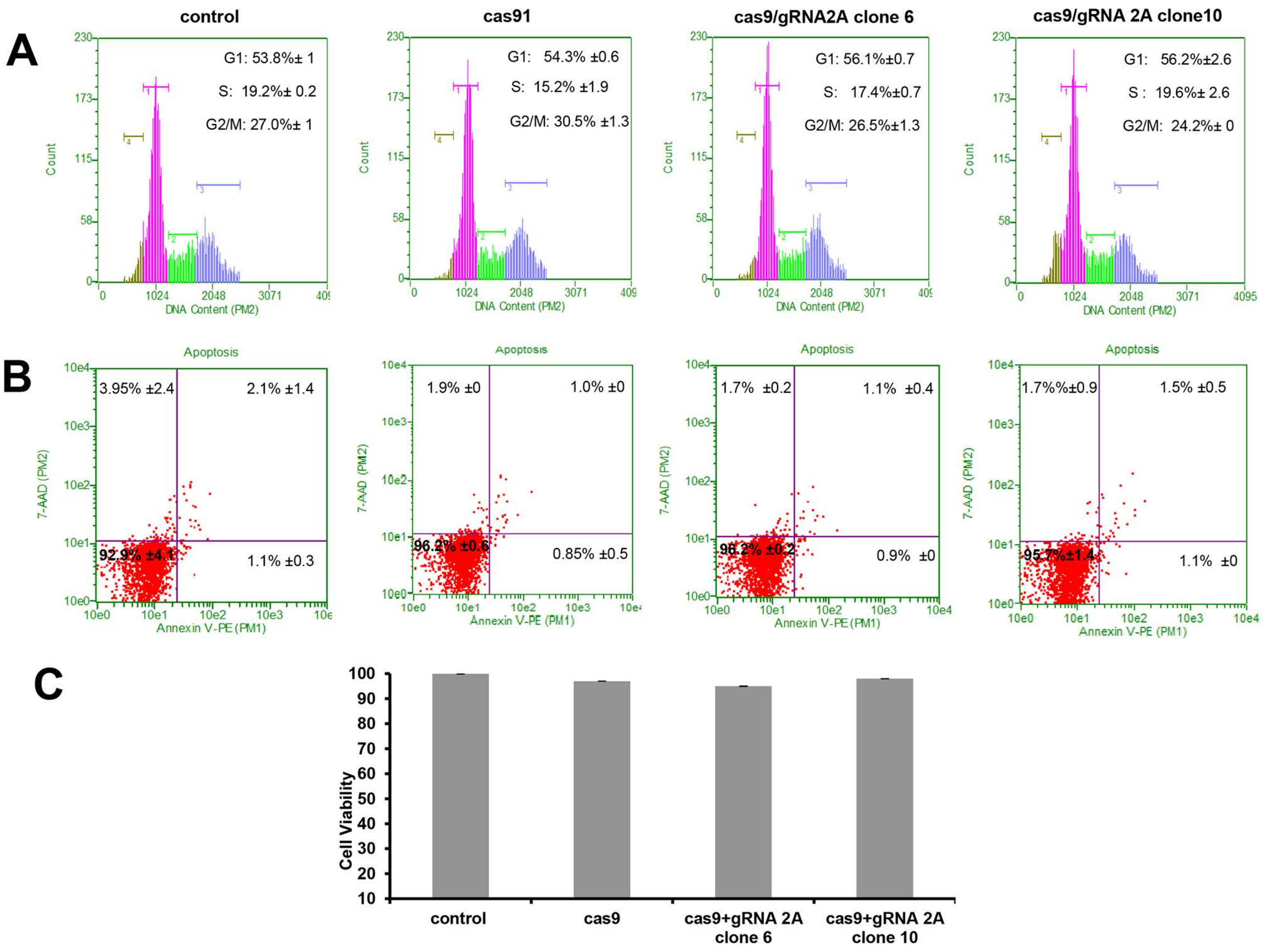
A
control
cas91
cas9/gRNA2A clone 6
cas9/gRNA 2A clone10
G1: 53.8%± 1
S: 19.2%± 0.2
G2/M: 27.0%± 1
G1: 54.3% ±0.6
S: 15.2% ±1.9
G2/M: 30.5% ±1.3
G1: 56.1%±0.7
S: 17.4%±0.7
G2/M: 26.5%±1.3
G1: 56.2%±2.6
S : 19.6%± 2.6
G2/M: 24.2%± 0
Count
DNA Content (PM2)
B
Apoptosis
7-AAD (PM2)
Annexin V-PE (PM1)
3.95% ±2.4
2.1% ±1.4
92.9% ±4.1
1.1% ±0.3
1.9% ±0
1.0% ±0
96.2% ±0.6
0.85% ±0.5
1.7% ±0.2
1.1% ±0.4
96.2% ±0.2
0.9% ±0
1.7%%±0.9
1.5% ±0.5
95.7%±1.4
1.1% ±0
C
Cell Viability
control
cas9
cas9+gRNA 2A clone 6
cas9+gRNA 2A clone 10

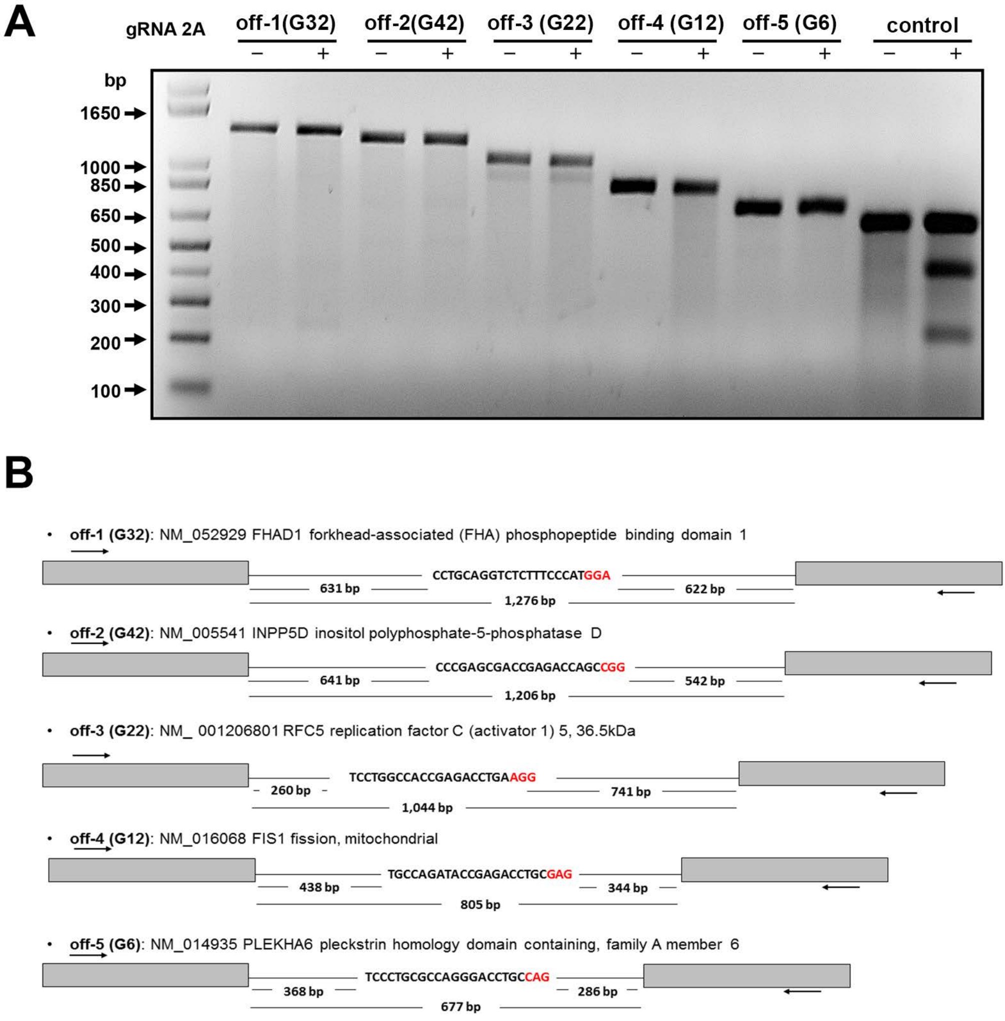

A
gRNA 2A
off-1(G32)
off-2(G42)
off-3 (G22)
off-4 (G12)
off-5 (G6)
control
− + − + − + − + − + − +
bp
1650
1000
850
650
500
400
300
200
100
B
• off-1 (G32): NM_052929 FHAD1 forkhead-associated (FHA) phosphopeptide binding domain 1
CCTGCAGGTCTCTTTCCCATGGA
631 bp
622 bp
1,276 bp
• off-2 (G42): NM_005541 INPP5D inositol polyphosphate-5-phosphatase D
CCCGAGCGACCGAGACCAGCCGG
641 bp
542 bp
1,206 bp
• off-3 (G22): NM_ 001206801 RFC5 replication factor C (activator 1) 5, 36.5kDa
TCCTGGCCACCGAGACCTGAAGG
260 bp
741 bp
1,044 bp
• off-4 (G12): NM_016068 FIS1 fission, mitochondrial
TGCCAGATACCGAGACCTGCGAG
438 bp
344 bp
805 bp
• off-5 (G6): NM_014935 PLEKHA6 pleckstrin homology domain containing, family A member 6
TCCCTGCGCCAGGGACCTGCCAG
368 bp
286 bp
677 bp

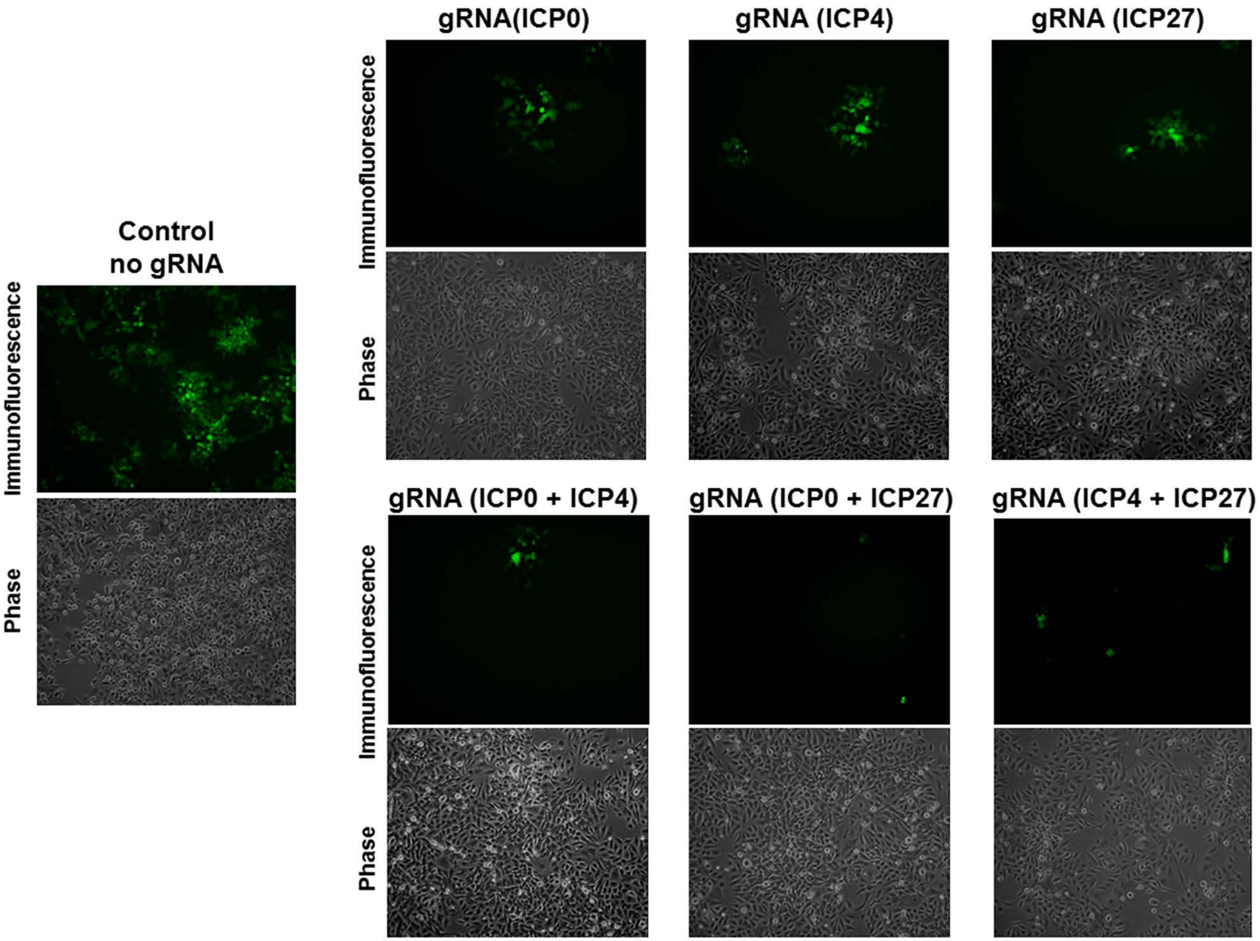
Control
no gRNA
Immunofluorescence
Phase
gRNA(ICP0)
gRNA (ICP4)
gRNA (ICP27)
gRNA (ICP0 + ICP4)
gRNA (ICP0 + ICP27)
gRNA (ICP4 + ICP27)
